# Multiple Pregnancy among Deliveries in a Tertiary Care Center: A Descriptive Cross-sectional Study

**DOI:** 10.31729/jnma.7897

**Published:** 2022-11-30

**Authors:** Rupa Bajagain, Chandrima Karki, Shilpi Mahato, Rachana Saha, Nitu Saha

**Affiliations:** 1 Department of Obstetrics and Gynaecology, Kathmandu Medical College and Teaching Hospital, Sinamangal, Kathmandu, Nepal; 2 Kathmandu Medical College and Teaching Hospital, Sinamangal, Kathmandu, Nepal

**Keywords:** *low birth weight*, *multiple pregnancy*, *preterm*

## Abstract

**Introduction:**

Multiple pregnancy is associated with increased obstetric complications as well as poor perinatal outcomes in developing countries because of the increased risk to both mother and baby. So better understanding of the risk factors is required to improve the quality of perinatal care. The aim of the study was to find out the prevalence of multiple pregnancies among deliveries in a tertiary care centre.

**Methods:**

A descriptive cross-sectional study was done among total deliveries in the Department of Obstetrics and Gynaecology of a tertiary care centre from inpatient records starting from 15 August 2020 to 15 February 2021 after obtaining ethical approval from the Institutional Review Committee (Reference number: 1208202005). Convenience sampling was done. Point estimate and 95% Confidence Interval were calculated.

**Results:**

Out of 4400 deliveries, multiple pregnancy was seen in 35 (0.79%) (0.53-1.06, 95% Confidence Interval).

**Conclusions:**

The prevalence of multiple pregnancies was similar to the studies done in similar settings.

## INTRODUCTION

Globally multiple pregnancies pose challenges to the Obstetrician. This is mainly due to increased maternal and foetal complications both in developed and developing countries. There has been a widespread increase in multiple pregnancies in recent years mainly due to late childbearing age, use of ovulation-inducing drugs and Assisted reproductive techniques (ART). The worldwide incidence of multiple pregnancies varies considerably and it is around 2-20 per 1000 births.^1^

Although major medical advances have improved the outcome of multiple births still they are associated with significant maternal and perinatal morbidity and mortality. Maternal and Perinatal morbidity and healthcare costs rise in multiple pregnancies because of associated prematurity, low birth weight (LBW), Hypertensive disorders, haemorrhage, increased operative interferences, and growth abnormalities.^2-3^ Compared to a singleton pregnancy, multiple pregnancies are reported to carry various complications.^3,4^ This keeps them in high vulnerable zone till later life. That is why a better understanding of the prevalence, risk factors and complications are often required to improve the quality of perinatal care and thereby decrease perinatal morbidity and mortality. In Nepal studies on multiple pregnancies and risk factors are very limited.

The aim of this study was to find out the prevalence of multiple pregnancies among deliveries in a tertiary care centre.

## METHODS

This descriptive cross-sectional study was conducted in the Department of Obstetrics and Gynaecology of Kathmandu Medical College and Teaching Hospital from 15 August 2020 to 15 February 2022 after obtaining ethical approval from the Institutional Review Committee (Reference number: 1208202005). It included all women admitted to the antenatal ward and labour room with clinical or ultrasound diagnosis of multiple pregnancies after 28 weeks of gestation. Convenience sampling was done. The sample size was calculated using the following formula:


$$\begin{array}{ll} \text{n} & ={\text{Z}}^{2}\times \frac{\text{p}\times \text{q}}{{\text{e}}^{\text{2}}}\\ & =1.96^{2}\times \frac{0.50\times 0.50}{0.02^{2}}\\ & =2401 \end{array}$$

Where,

n= minimum required sample size
Z= 1.96 at 95% Confidence Interval (CI)
p= prevalence taken as 50% for maximum sample size calculation
q= 1-p
e= margin of error, 2%

The calculated sample size was 2401, by adding 10% non-response rate, the sample size was 2668. However, the final sample size taken was 4400. Informed consent was taken from each participant by explaining the objectives of the research. They were followed from admission through delivery up to discharge of both mother and baby from the hospital. A pretested semistructured proforma was used for data collection. Data related to maternal age, parity, chorionicity, maternal medical and obstetrical complications, mode of conception, mode of delivery, postpartum complications, the neonatal outcome in terms of birth weight, Appearance, Pulse, Grimace, Activity, and Respiration (APGAR) score, NICU admission and perinatal death were taken into account.

Data were entered in an excel sheet and analysed with IBM SPSS Statistics 20.0. Point estimate and 95% CI were calculated .

## RESULTS

**Figure 1:**
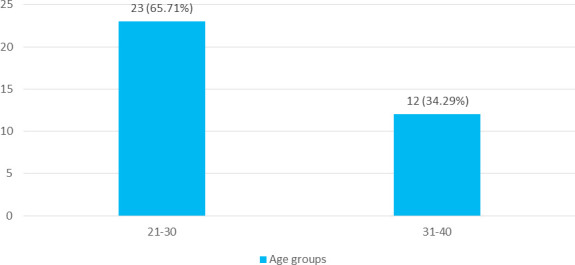
Age Distribution (n= 35).

Among 4400 deliveries, multiple pregnancy was seen in 35 (0.79%) (0.53-1.05, 95% CI). The distribution of multiple pregnancies in relation to maternal sociodemographic profile is shown below. About 23 (65.71%) of pregnant women were between the age group 21-30 years, whereas 12 (34.29%) of women were in the age group 31-40 years (Figure 1).

**Figure 2:**
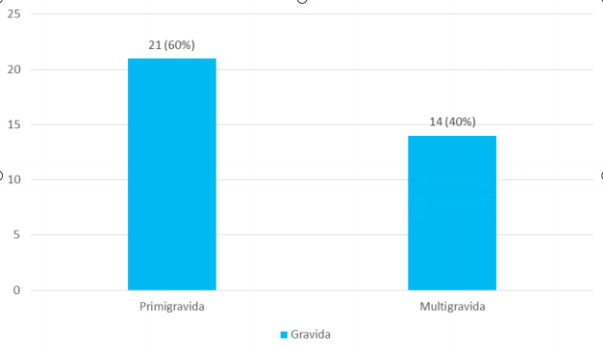
Parity of mothers (n= 35).

Among them, the majority of the women were primigravida 21 (60%) in this study (Figure 2).

Out of 35 cases of multiple pregnancies, 11 (31.43%) were at 30-34 weeks period of gestation at the time of delivery whereas 19 (54.29%) had delivered at 34-37 weeks of gestation. Only 4 (11.43%) had a delivery at term (Table 1).

**Table 1:** Maternal period of gestation (POG) (n= 35).

POG (weeks)	n (%)
<30	1 (2.86)
30-34	11 (31.43)
34-37	19 (54.29)
>37	4 (11.43)

**Figure 3:**
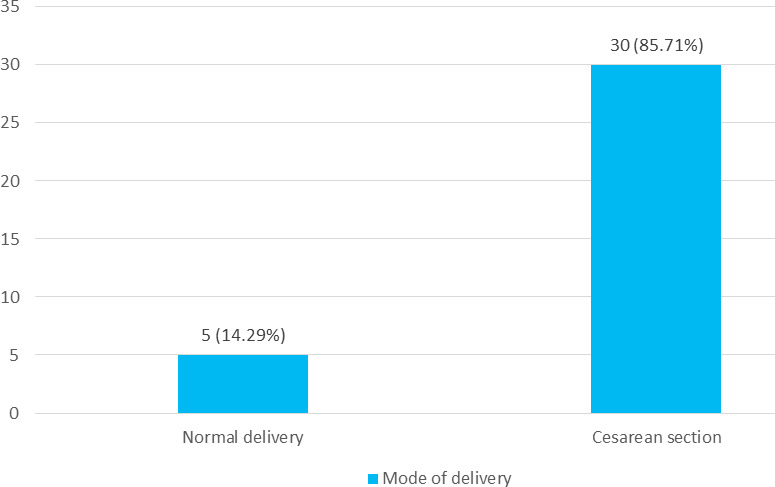
Mode of delivery (n= 35).

Among 35 cases of multiple pregnancies, spontaneous vaginal delivery was seen in 5 (14.29%) of twins whereas 30 (85.71%) of cases underwent caesarean section. The most common indication for caesarean section was PPROM and malpresentation. Other indications were cephalopelvic disproportion, severe pre-eclampsia and discordant twins (Figure 3).

In this study the mode of conception was also taken into account which shows that 26 (74.28%) of women conceived spontaneously, ovulation induction was done in 3 (8.57%) and Intrauterine insemination and In Vitro fertilisation were done in 3 (8.57%) of the cases. Also regarding chorionicity, this study shows that diamniotic dichorionic twins constitute about 20 (57.14%) while diamniotic monochorionic (DAMC) twins constitute about 11 (31.43%). There were 3 (8.57%) monoamniotic monochorionic (MAMC) twins.

In this study, maternal complications such as premature rupture of the membrane were 14 (40%), hypertensive disorder of pregnancy 6 (17.1%), and thyroid disorder in pregnancy constituted 5 (14.28%). Preterm labour was the most common complication in this study comprising 31 (88.73%). Post partum haemorrhage was found in 2 (5.71%). Foetal complications included intra uterine growth restriction (IUGR) in 5.7% in the first twin and 11.42% in the second twin, birth weight discordance in 7.04% and neonatal death at 5.71% in the first twin and 17.1% in the second twin and 100% (one out of one) in a triplet. After delivery, all babies were taken to the NICU. Prolonged NICU admission was required for preterm and IUGR babies. Only 15.49 % of babies had normal birth weight among 70 live births.

**Table 2:** Maternal complications in multiple pregnancy (n= 35).

Maternal complications	n (%)
Diabetes Mellitus	4 (11.42)
Pre eclampsia	1 (2.86)
Thyroid disorder	5 (14.28)
Premature rupture of membrane	14 (40)
Postpartum haemorrhage	2 (5.71)
Anaemia	2 (5.71)
Preterm labour (< 37weeks)	31 (88.57)
Hypertensive disorder	6 (17.14)
Diabetes Mellitus	4 (11.42)

## DISCUSSION

The prevalence of multiple pregnancies in this study was 0.79% which is similar to studies done in Nigeria (2.21-3.25%).^5,6^ Among the pregnant mothers included in this study, the majority were between the ages of 21-30 years. Other studies observed similar ages in their study as well.^7-8^ Incidence of twins is higher in primigravida 21 (60%) in this study. However, in a study done in India, it was observed in 59% nulliparous with less incidence in multiparous females.^9^ In this study about 74% of females conceived spontaneously, 8% were induced with an ovulation-inducing agent and 9% were IVF-conceived pregnancies. In another study done in India, only 17.3% were reported to have had ovulation induction.^10^

Multiple pregnancies are associated with greater complications in both mother and fetus. Among the maternal complication, preterm delivery was the major problem faced. Among the 35 cases of multiple pregnancies, 31 (88.57%) mothers delivered preterm. Preterm delivery was seen to be more in the African population. In studies done in Nigeria, the incidence was observed to be 41% and 39.7% respectively.^5-6^ Another study reported that preterm delivery occurs in 50% of twins and 10-12% of preterm deliveries are constituted by twins.^11^ All were spontaneous onset preterm labour followed by prelabour premature rupture of membrane (PPROM) 40%, medical disorders in pregnancy like gestational diabetes mellitus 11.42%, hypertensive disorder comprises 17.14% of which preeclampsia occurred in 2.86%. A high incidence of hypertensive disorder was also observed in another study at 18% and 22.6% in other studies.^12,13^ Other complications were anaemia (5.71%) and postpartum haemorrhage (5.71%).

There were a total of 70 live births and one stillbirth among the 35 cases of multiple pregnancies. Among them, only 15.49% of babies had normal weight. The low birth weight means the weight of the baby was less than 2.5 kg. The mean birth weight of the first twin was 1.85±0.56 kg and for the second twin was 1.88±0.56 kg the neonatal death rate was 5.71% for the first twin and 17.1% for the second twin in this study. This was comparable with the study done in India (17.5%).^12^ This may be due to the fact that our centre is a tertiary care centre with good neonatal facilities.

This is a single centered study with limited sample size. There is no control in this study. Also the study is conducted during the pandemic of covid 19 where people feared to visit hospital hence leaving space for bias.

## CONCLUSIONS

The prevalence of multiple pregnancies among total deliveries was found to be similar to other studies done in a similar setting. Multiple pregnancies were common in primigravida. Maternal complications include preterm labour, hypertension, PPROM, postpartum haemorrhage and diabetes. Caesarean section is the commonest mode of delivery. Prematurity and intrauterine growth restriction are the commonest foetal complications. So better understanding of the different factors, proper antenatal care, planned delivery and better facilities is required to improve the quality of perinatal care.
